# Strategies for delivery of CRISPR/Cas-mediated genome editing to obtain edited plants directly without transgene integration

**DOI:** 10.3389/fgeed.2023.1209586

**Published:** 2023-07-20

**Authors:** Zuzana Kocsisova, Viktoriya Coneva

**Affiliations:** CTC Genomics, Centro de Tecnologia Canavieira, Saint Louis, MO, United States

**Keywords:** transgene-free, genome editing, plants, CRISPR, transformation

## Abstract

Increased understanding of plant genetics and the development of powerful and easier-to-use gene editing tools over the past century have revolutionized humankind’s ability to deliver precise genotypes in crops. Plant transformation techniques are well developed for making transgenic varieties in certain crops and model organisms, yet reagent delivery and plant regeneration remain key bottlenecks to applying the technology of gene editing to most crops. Typical plant transformation protocols to produce transgenic, genetically modified (GM) varieties rely on transgenes, chemical selection, and tissue culture. Typical protocols to make gene edited (GE) varieties also use transgenes, even though these may be undesirable in the final crop product. In some crops, the transgenes are routinely segregated away during meiosis by performing crosses, and thus only a minor concern. In other crops, particularly those propagated vegetatively, complex hybrids, or crops with long generation times, such crosses are impractical or impossible. This review highlights diverse strategies to deliver CRISPR/Cas gene editing reagents to regenerable plant cells and to recover edited plants without unwanted integration of transgenes. Some examples include delivering DNA-free gene editing reagents such as ribonucleoproteins or mRNA, relying on reagent expression from non-integrated DNA, using novel delivery mechanisms such as viruses or nanoparticles, using unconventional selection methods to avoid integration of transgenes, and/or avoiding tissue culture altogether. These methods are advancing rapidly and already enabling crop scientists to make use of the precision of CRISPR gene editing tools.

## 1 Introduction

### 1.1 Editing plants for research, energy, and food

Humankind’s radically increased ability to deliver precise genotypes in crops thanks to advances in breeding, transformation, transgenics, and editing is helping growers keep pace with increased demand for food and energy. One could say the primary limitation to crop improvement is no longer, “can we make a precise sequence change in a precise location in eukaryotic genomic DNA?” but rather “which change in the DNA will have the desired impact on a crop’s phenotype”. In many crops, genome engineering techniques have enabled researchers and breeders to take advantage of a vast knowledge base of plant physiology, pathology, and genetics. This knowledge base is far from complete for all useful traits in all crops, with the majority of studies and commercial applications focusing on model species or widely grown, annual crops. Gene editing tools are being applied in basic research to close the knowledge gap, but the requirements for gene editing techniques for agriculture are different than those for research. The process can be less efficient overall, since a larger team can produce many plants from which to select the elite edited plant for commercialization. However, the technique must invariably deliver a precise result that is stable and safe for the environment and the consumer.

While plant transformation techniques for creating transgenic varieties in specific crops and model organisms are quite advanced, the major obstacles to applying gene editing technology in most crops are reagent delivery and plant regeneration (Altpeter et al., 2016). Recalcitrance to transformation is a fundamental barrier to realizing the technology in many species and cultivars. Much progress has been reported recently towards overcoming recalcitrance (Kamm et al., 2019; Debernardi et al., 2020). Typical plant transformation protocols to produce transgenic, genetically modified (GM) varieties rely on tissue culture, transgenes, and chemical selection. Typical protocols to make gene edited (GE) varieties also use transgenes, even though the transgenes may be undesirable in the final crop product. For this reason, the techniques that are effective for crops such as maize or tomato may be impractical for crops with long generation time, that are vegetatively propagated, reproduce through apomixis, are polyploid, self-incompatible, and/or highly heterozygous (May et al., 2023). Desirable allele combinations in crops such as grape, potatoes, or sugarcane would be lost due to allele segregation during meiosis (Veillet et al., 2019; Krishna et al., 2023). Waiting 10–50 years per generation, as is the case in trees or bamboo for example, is prohibitive to many crop improvement projects (Ramirez-Torres et al., 2021; Anders et al., 2023). As a result, there is a need for techniques which can precisely modify a genome without relying on tissue culture, transgenics, and breeding away transgenic reagents.

Within the field of gene editing, researchers are moving beyond single gene knockout traits, adding in precise templated editing, multi-gene editing, and integrating gene editing into breeding. For a comprehensive review, see (Lyzenga et al., 2021) and (Caradus, 2023). In these more complex applications, breeding away a transgene may be undesirable, impractical, or statistically improbable even in crops where breeding is performed routinely. Furthermore, even once a transgene has been removed by breeding, there remain implications of that transgene in the plant’s history, particularly in jurisdictions with regulation centered around the delivery method rather than the final product (Sprink et al., 2016; Gupta et al., 2021; Turnbull et al., 2021; Caradus, 2023). If methods for transgene-free delivery of editing reagents become more efficient, these methods may turn out to be more convenient in most crops.

### 1.2 CRISPR-based tools for editing plant genes

While gene editing refers to a variety of tools, in this review we will focus on CRISPR-based methods. For a more comprehensive review of general gene editing methods in plants, see (Qi, 2019). The common feature of CRISPR-based methods is the use of a multi-purpose programmable editor protein combined with one or more RNA molecules which guide the complex to a precise location in the genome. The protein is typically an endonuclease which creates a double-stranded break in the genome. The double strand break is repaired imprecisely (e.g., by non-homologous end joining) for a knockout edit (typically a small deletion, insertion, or substitution) or precisely (e.g., by homology-directed repair or prime editing) for a templated edit. The nuclease is typically one of the forms of Cas9, but Cas12a is also used frequently, and other CRISPR-associated nucleases are available. In each case, the active gene editor is a complex of protein and RNA. The RNA is composed of an approximately 20 nt region used to find a match in the target DNA—to “program” the editor—and of a structural component (called a scaffold and/or tracrRNA, which can be a separate molecule). The fraction of the genome within reach of the gene editor is limited only by the availability of a protospacer-adjacent motif and possibly chromatin accessibility (Liu et al., 2019a; Jain et al., 2021). Other gene editors exist which consist of only proteins such as TALENs and ZFNs (Bibikova et al., 2002; Christian et al., 2010; Carroll, 2011; Cermak et al., 2011). Since the invention of CRISPR-based gene editing in 2012, the field has exploded with more advanced gene editors that allow wider access to the genome or better precision of editing (Jinek et al., 2012; Capdeville et al., 2023). Notably, researchers modified Cas9 into a nickase and fused it to other functional domains to create base editors, prime editors, etcetera (Komor et al., 2016; Anzalone et al., 2019).

### 1.3 The bottlenecks limiting transgene-free gene editing in plants

To optimize a process, it is helpful to understand what success looks like (see Supplementary Note S1), be able to measure outcomes (see Supplementary Note S2) and understand its bottlenecks. In the case of plant gene editing, successful recovery of a gene edited plant requires that the following key processes all take place successfully. First, **deliver** reagents to a cell. In plants, the rigid cell wall is a major barrier (Laforest and Nadakuduti, 2022). Next, CRISPR reagents bind DNA and **cut** (make a double strand break or single-strand nick). Then the cell’s machinery (or delivered machinery) **repairs** the break or nick, possibly using a co-delivered template. Next the cell with this new edit **regenerates** into a new plant. Finally, we **identify** this new plant as edited and separate it from other non-edited plants. The overall probability of success can be calculated by multiplying these probabilities: P(success) = P(deliver) x P(cut) x P(repair) x P(regenerate) x P(identify).

The main distinguishing steps to achieve transgene-free editing are in how reagents are delivered (i.e., whether as DNA, RNA, or protein) and how the edited plant is recovered (whether any form of selection is used to kill cells which did not receive reagents). These are described in the sections below.

## 2 Results and discussion

### 2.1 Step 1: deliver chosen cargo using chosen vehicle

**FIGURE 1:**
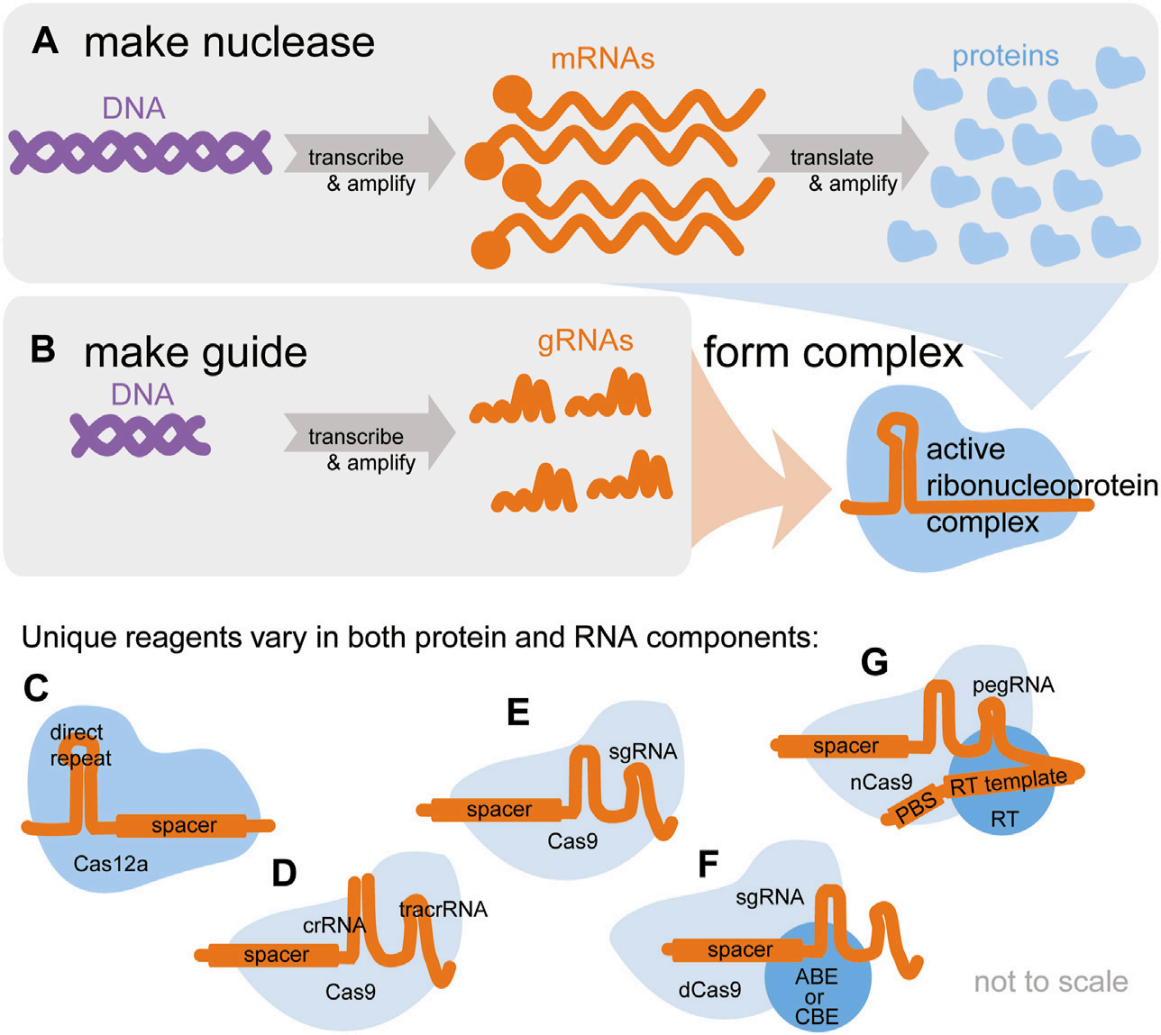
Active CRISPR gene editors are ribonucleoproteins, complexes of an editor nuclease and a guide RNA. Both components must be delivered for editing to take place. **(A)** The central dogma describes how CRISPR nuclease reagents originating in DNA are transcribed and amplified into mRNA and translated and amplified into protein. **(B)** The editor is programmed by a guide RNA (gRNA), which is also transcribed and amplified from DNA. **(C–G)** A multitude of CRISPR-based gene editors is available. Some vary only in the protein nuclease component, others only in the gRNA component, and some in both. **(C)** Cas12a nucleases are programmed by a single crRNA. **(D,E)** Cas9 nucleases are typically programmed by a crRNA annealed to a tracrRNA **(D)** or a single chimeric sgRNA **(E)**. **(F)** Base editors require a special fusion protein and can be programmed with a sgRNA or separate crRNA + tracrRNA. **(G)** Prime editors require a special fusion protein and are programmed with a special pegRNA. Researchers must ensure the exact desired editing reagents are available in the chosen format (DNA, RNA, or protein).

Reagent delivery is a major challenge to plant genetic modification and gene editing alike (Altpeter et al., 2016; Mao et al., 2019). The first step for successful gene editing is to prepare the GE reagents (cargo) and deliver them into plant cells using an appropriate method (vehicle). The GE reagents can be delivered in the form of DNA, similar to transgenes, and then transcribed and translated using the cell’s machinery. However, unlike transgenes, the GE reagents can also be delivered as RNA, protein, or a mixture of those “central dogma” components, meaning the researcher must make a choice regarding which to use (Figures 1A, B). DNA has advantages: it is stable, and each molecule of DNA can produce many molecules of RNA and protein. However, DNA is inactive and relies on the cell’s machinery to produce active gRNA and protein complexes. RNA and protein are less stable, but ribonucleoproteins have the advantage of being “ready-to-edit”. Detailed considerations regarding cargo choice are described in the next section.

While some gene editing reagents can edit DNA in a tube directly and easily, GE reagents do not enter plant cells in a straightforward way. The cell membrane and cell wall are the primary barriers to entry and must be bypassed using some delivery vehicle. The vehicle for delivering this cargo must be chosen to match the explant and the desired cargo (Table 1). The considerations regarding vehicle choice are described in the section below.

**TABLE 1:** Guide to choosing compatible reagent (cargo) and delivery method (vehicle) for transgene-free gene editing.

	**Cargo:**	DNA	mRNA	gRNA	Protein
		**Pros:** Nuclease & guide delivered in same format, stable; easy & inexpensive to prepare & customize; inherent amplification by transcription & translation	**Pros:** Avoid DNA; compatible with vehicles for nucleic acids; inherent amplification	**Pros:** Avoid DNA (typically co-delivered with nuclease in the form of mRNA or protein	**Pros:** Avoid DNA; ready-to-edit ribonucleoproteins; small
		**Cons:** Typically transgenic, requires host cell transcription & translation	**Cons:** gRNA is delivered separately, less stable, more expensive to prepare & customize	**Cons:** Nuclease is delivered separately or complexed (RNPs); also see cons for protein and mRNA	**Cons:** gRNA is delivered separately or complexed (RNPs); not compatible with all delivery vehicles for nucleic acids; more expensive to prepare & customize
**Vehicle:**					
*Agrobacterium*	**Pros:** long-established in the field & scalable to production pipelines	Well established	Not determined	Not determined	Plausible with new PT3SS strains (Raman et al., 2022)
	**Cons:** typically transgenic; many genotypes are recalcitrant				
Particle bombardment	**Pros:** long-established in the field; can deliver RNPs	Well established	Demonstrated (Zhang et al., 2016)	Established (Svitashev et al., 2016; Liang et al., 2017)	Established (Martin-Ortigosa and Wang, 2014; Svitashev et al., 2016; Liang et al., 2017; Poddar et al., 2023)
	**Cons:** may damage chromosomes; harder to scale up to production pipeline				
Nanocarriers (various)	**Pros & Cons** difficult to generalize across carrier types	Demonstrated (Demirer et al., 2019; Demirer et al., 2021)	Demonstrated (Demirer et al., 2021)	Demonstrated (Demirer et al., 2021)	Not determined
	**Pros:** promise for tissue-culture-free and/or explant-independent delivery				
	**Cons:** individual carriers are usually optimized for a single cargo type; not well established				
Viruses (various)	**Pros:** systemic virus can spread throughout the plant; promise for tissue-culture-free delivery	Demonstrated see review (Laforest and Nadakuduti, 2022)	Demonstrated see review (Laforest and Nadakuduti, 2022)	Demonstrated see review (Laforest and Nadakuduti, 2022)	Not determined
	**Cons:** cargo size limitation; host range limitation; exclusion from germline				
Protoplasts	**Pros:** very efficient DNA-free delivery	Well established	Demonstrated (Stoddard et al., 2016)	Established (Woo et al., 2015)	Established (Woo et al., 2015)
	**Cons:** challenging to regenerate, not yet demonstrated in most species; risk of tissue culture effect				
Zygotes	**Pros:** avoid cell wall, but straightforward regeneration	Demonstrated (Toda et al., 2019)	Not determined	Demonstrated (Toda et al., 2019)	Demonstrated (Toda et al., 2019)
	**Cons:** technically challenging, narrow time window; extensive species-specific optimizations				
Pollen (HI-Edit)	**Pros:** fits into existing haploid induction systems	Demonstrated (Kelliher et al., 2019)	Not determined	Not determined	Not determined
	**Cons:** short window for GE reagent expression; may produce mosaics; limited by availability of haploid induction system & tools; species-specific optimization				
Grafting	**Pros:** straightforward to apply in many dicots	NO	Demonstrated (Yang et al., 2023)	Demonstrated (Yang et al., 2023)	Not determined
	**Cons:** produces mosaics, currently restricted to dicots; requires a graft-compatible transgenic rootstock				

In addition to feasibility of delivery (matching the cargo to the vehicle to the explant), researchers must also ensure their desired editing reagents are available in the chosen format (Figures 1C–G). Once the cargo and vehicle are chosen, and the reagents are delivered to the target cell(s) - usually a quick process measured in milliseconds or hours, the researcher must recover the edited plant, which can be straightforward or involved and can take days, weeks, or months. Methods for edited plant recovery are described in step 2. The choices of cargo and vehicle have a direct impact on the feasibility and efficiency of transgene-free gene editing outcomes.

#### 2.1.1 Choose the cargo

##### 2.1.1.1 DNA cargo

**FIGURE 2:**
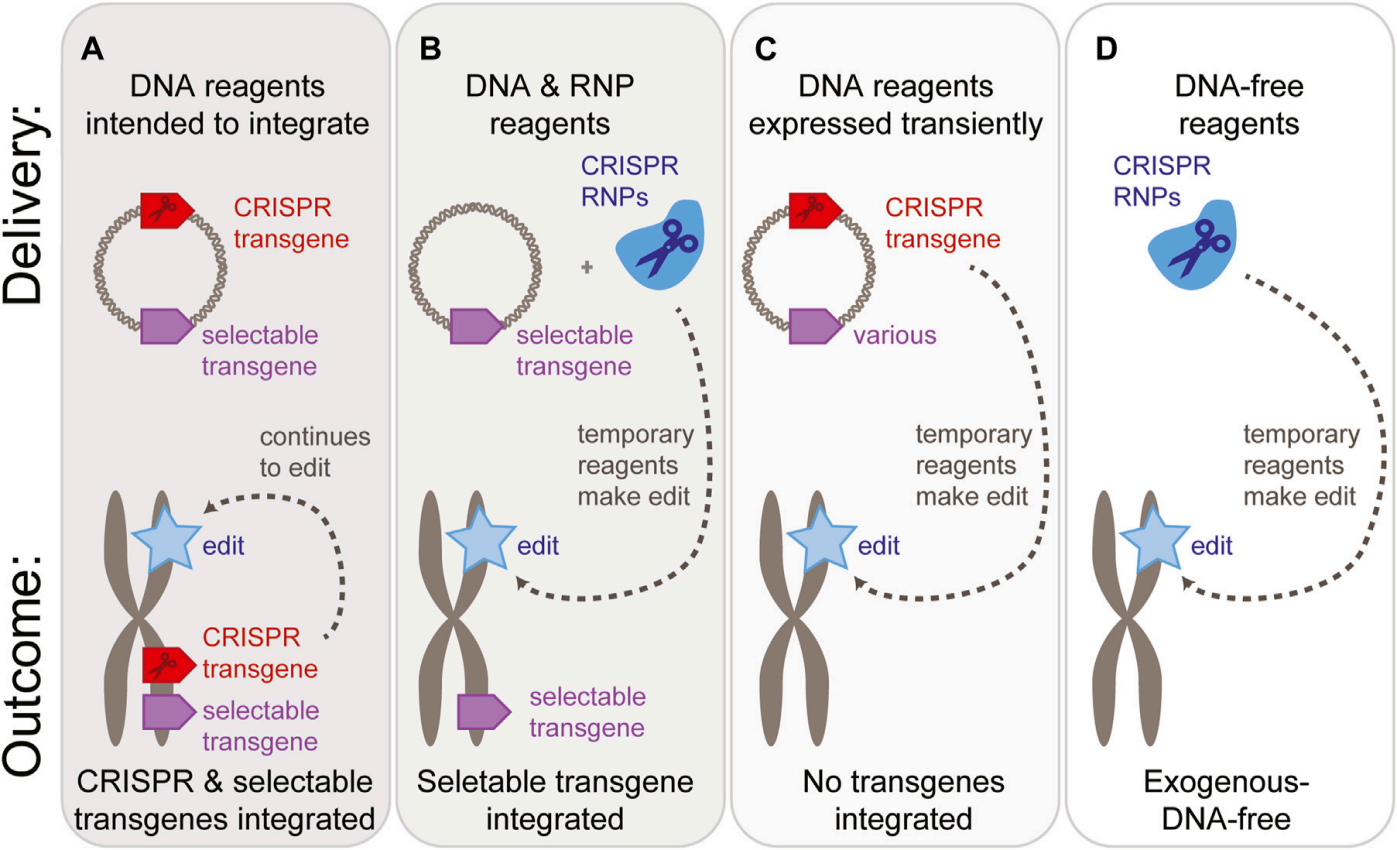
Reagent delivery options and possible transgene status of the resulting plants. A “gray area” exists between fully transgenic, CRISPR-expressing plants **(A)** & exogenous-DNA-free methods **(D)**. There may be applications where an integrated selectable marker transgene is acceptable, as long as the CRISPR reagents are no longer present **(B)**. Alternatively, delivery of DNA-based reagents may be acceptable as long as no transgenes are present in the final plant **(C)**.

DNA is easy to work with, stable, and has been the focus of delivery methods for transgenics for decades. The first studies to demonstrate CRISPR gene editing in plant cells delivered DNA (Figure 2A) (Nekrasov et al., 2013; Shan et al., 2013; Li et al., 2013; Feng et al., 2013; Xie and Yang, 2013; Miao et al., 2013). Historically, plant cell delivery mechanisms focused on delivery of DNA; thus a wide range of options is available [reviewed in Ghogare et al. (2021) and Kausch et al. (2021)]. Some are described below in the section on vehicle choice. Some of these methods can only deliver DNA in a particular form, such as a single-stranded DNA excised from a circular plasmid, while others can accommodate variations in stranded-ness, length, and chemical modifications.

Most often, CRISPR reagents are prepared on a circular dsDNA plasmid using common cloning methods in *E. coli*. The nuclease and gRNA components can be prepared on separate plasmids, an approach which offers ease of mixing-and-matching. Alternatively the nuclease and gRNA(s) can be on a single plasmid, ensuring that they are co-delivered. Each can be expressed from its own promoter, or a single transcript unit can be used to express the nuclease and gRNA(s). When multiple gRNAs are needed, these can be expressed from separate promoters (often pol-III promoters such as pU3 or pU6), or from a multiplex array with tRNAs, self-cleaving ribozymes, or Cas12a-proccesed crRNAs expressed from pol-II promoters (such as pUBI). For a review of plasmid design considerations, see (Hassan et al., 2021). The purified plasmid(s) can be delivered using several of the vehicles described below in the section on vehicle choice. Alternatively, the plasmid can be delivered to *Agrobacterium* or a virus, which then deliver a portion of the plasmid to the plant cell (Gelvin, 2003; Liu et al., 2023). Some of these delivery vehicles are size-limited or require particular sequence elements, so it is important to consider the delivery vehicle during the design of the plasmid.

DNA is also a very useful cargo to deliver DNA templates for homology-directed repair (Huang and Puchta, 2019). These templates can also be on a circular dsDNA plasmid, or they can be delivered as a linear dsDNA PCR product or a ssDNA. One advantage of non-plasmid DNA is to allow chemical modifications that can aid the probability of homology-directed versus random blunt-ended integration. If a DNA template needs to be delivered, this may influence the decision towards also delivering nuclease and gRNA as DNA so that the reagents can be combined and co-delivered. However, it is not a requirement, and DNA templates can be co-delivered with mRNA and/or ribonucleoproteins.

Additionally, consider whether the process will employ a transgene as a visual marker, selectable marker, or additional trait. These transgenes could be delivered as DNA while the CRISPR reagents are delivered as mRNA or ribonucleoproteins (Figure 2B). Alternatively, it may be practical to deliver CRISPR reagents in the same DNA format, most often on the same plasmid (Figures 2A, C).

When reagents are delivered as DNA, the reagents can be expressed from their extra-chromosomal form. This is often referred to as “transient” expression (Figure 2C). The DNA can remain in an extra-chromosomal form for minutes, hours, or days (Tyurin et al., 2020). At some point after reaching the nucleus, a subset of the DNA reagents integrate into the genome by a variety of mechanisms that are only partially understood. If these reagents integrate into a chromosome, the transgenes will begin to replicate as part of the plant’s own genome, and if the genomic position is suitable, the transgenes will be expressed. Typically, this is referred to as “stable” expression.

The considerations for DNA delivery methods for transgene-free gene editing are different, and in fact opposite compared to transgenics. The ideal DNA delivery platform for transgenics results in a small amount of extra-chromosomal DNA, of which a large proportion integrates at low-copy. The ideal DNA delivery platform for gene editing results in a large amount of highly expressing extra-chromosomal DNA, of which a very small proportion (preferably none) integrates. Note that in scientific literature the term “transient” is often used as a proxy for extra-chromosomal and the term “stable” is used as a proxy for chromosomally integrated transgenes (Kim et al., 2010). Integration of extra-chromosomal DNA is essentially a one-way process, therefore researchers who are hoping to avoid making a transgenic plant using DNA-based delivery usually seek to avoid integration in the first place. However, as described below in the section on full transgenic selection, a transgene can be removed through either excision or through segregation during meiosis, and either option could be followed with selection against the transgene.

The main advantages of DNA cargo are the flexible in-house design of the reagent, DNA’s stability, the ability to deliver greater amounts over time by virtue of transcription and translation, and compatibility of DNA with a wide range of delivery vehicles. The main limitations of DNA cargo are that it must be transcribed and translated by the host cell, and, for transgene-free applications, DNA reagents present a challenge due to their propensity to integrate into the plant’s genome.

##### 2.1.1.2 RNA cargo

The clearest way to avoid transgenes is to employ delivery methods without exogenous DNA (Tsanova et al., 2021; Poddar et al., 2023), often termed less precisely as “DNA-free” methods (Figure 2D). In practice, the available cargo is either RNA or protein. Since these reagents are only available for a short time, both theorizing and empirical evidence point to a lower likelihood of off-target editing or unwanted reagent integration into either the targeted locus or random locations in the genome (Stoddard et al., 2016; Liang et al., 2017).

Delivery of gRNA and mRNA encoding a nuclease to plant cells has been reported by only a few groups, suggesting this method remains challenging and non-trivial to reproduce (Stoddard et al., 2016; Zhang et al., 2016; Imai et al., 2020). However, mRNA-based delivery is mainstream in mammalian tissue culture, as demonstrated by suppliers offering off-the-shelf mRNA versions of a range of gene editing reagents. Since RNA is a nucleic acid similar to DNA, many of the same delivery methods can be used, including protoplast transfection, gene gun particle bombardment, and appropriately selected nanocarriers, as described below in the section on vehicle choice. As illustrated in Figures 1A, B, delivery of mRNA means the reagents do not require cellular machinery for transcription, however, the delivery dose may need to be adjusted to account for the loss of amplification by transcription. For example, the Voytas lab found that expression of a fluorophore from mRNA was barely visible and editing by TALENS delivered as mRNA was ∼12-fold lower than with DNA delivery (Stoddard et al., 2016). Researchers must also consider that RNA is typically less stable than DNA, and that the gRNA must “wait” for the mRNA encoding nuclease to be translated into protein (Zhang et al., 2016). If synthetic gRNA is used, it can be chemically stabilized in a variety of ways (Kelley et al., 2016; Allen et al., 2021; Rozners, 2022). mRNA can be stabilized through the design of stabilizing 5′ and 3′ UTRs, 5′ caps, and appropriate poly-A tails (Stoddard et al., 2016). One reported advantage of RNA-based delivery is the observation of significantly fewer random insertions compared to a DNA-based plasmid (Stoddard et al., 2016).

The main advantages of RNA cargo are avoiding DNA, but still employing a nucleic acid. Since RNA reagents are typically produced from a plasmid, this method offers the same benefits of customizability, although the *in-vitro* transcription must be performed separately for each new reagent. RNA-based delivery has an extremely low risk of integrating reagents into the genome. Another advantage is the ability to deliver modified bases, for example, to target polymorphic sequences (Krysler et al., 2022). Due to its chemical similarity to DNA, several delivery vehicles that were designed for DNA can be adapted to RNA with only minor modifications. The main limitations of RNA cargo are that RNA is less stable than DNA and that RNA is typically synthesized or produced by *in-vitro* transcription, which is an additional expense compared to DNA delivery. In addition, the mRNA lacks the amplification step of transcription, so it is likely that a higher dose must be delivered for a comparable effect to DNA delivery.

##### 2.1.1.3 Protein cargo

Functional gene editors are complexes of gRNA and protein (ribonucleoproteins, RNPs, Figure 2), and many groups have demonstrated success in delivering these “ready-to-edit” reagents [Svitashev et al., 2016; Liang et al., 2017; Poddar et al., 2023 and many others, reviewed in Zhang et al. (2021)]. RNPs require neither transcription nor translation machinery from the host cell, although this does mean that a sufficient quantity must be delivered. The complexes are easily assembled in a simple aqueous buffer at ambient temperature in a matter of minutes, requiring nothing more than pipetting. The protein can be produced and purified in-house or obtained commercially. While purification of nuclease proteins is relatively routine, it is not yet trivial to obtain nuclease fusions (e.g., base editors, prime editors, etc.—see Figure 1C) thereby presenting a current limitation to the use of this technology for applications beyond the creation of random indels. gRNAs can be produced by *in-vitro* transcription in-house or commercially synthesized. While RNA is generally less stable than DNA, gRNAs are less prone to premature degradation when they are protected within the nuclease. Nevertheless, RNA modifications can be employed to stabilize the gRNAs (Poddar et al., 2023). Ribonucleoproteins can be useful in rapid screening to identify the best gRNA in a transient protoplast assay, for example, because new gRNAs can be purchased and tested without time-consuming steps to clone new gRNAs into plasmids.

The delivery vehicles for proteins are somewhat limited compared to those for nucleic acids. Particle bombardment into intact plant cells and PEG/Ca^2+^-mediated transfection into protoplasts are most common, although other methods are described below in the section below on vehicle choice. The protein component is more sensitive to denaturation, which means that the prepared RNPs must be maintained within a narrower range of temperature, pH, salinity, etcetera compared to DNA and RNA.

The main advantages of ribonucleoprotein cargo are that these reagents are ready-to-edit and that this is an approach entirely free of exogenous DNA, thus without risk of transgenes. The main limitations of ribonucleoprotein cargo are the difficulty in obtaining the purified protein of a custom gene editors depicted in Figures 1F, G and delivering a non-denatured RNP in sufficient quantity using a limited number of delivery vehicles.

##### 2.1.1.4 Match reagents to desired editing outcome

The reagents must be matched to the desired outcome of gene editing. For simple single-locus knockout editing by double-strand break and non-homologous end joining, it is sufficient to deliver the appropriate nuclease with the appropriate gRNA, which can be accomplished in a DNA-free way using ribonucleoproteins (Woo et al., 2015; Svitashev et al., 2016). For larger deletions or when navigating genomes with polymorphisms between copies of a gene, a nuclease and multiple gRNAs may need to be delivered (Eid et al., 2021). Similarly, for base editing, the appropriate base editor and gRNA must be delivered. In all designs employing Cas9 and similar nucleases, one must consider whether a crRNA + tracrRNA complex or a sgRNA is more appropriate and cost-effective to synthesize. Avoiding double strand breaks offers an advantage in product purity—i.e., resulting alleles are more likely to be identical to each other rather than a mixture of small deletions/insertions/substitutions (Xie et al., 2022).

For more precise sequence replacement, an appropriate template must be co-delivered. If the template is in the form of DNA, this may influence the decision whether the remaining reagents are also delivered as DNA, or whether advantages remain to DNA-free delivery methods. Several characteristics of the DNA template can influence the choice of delivery method. Is the DNA template double-stranded or single-stranded? How long is the DNA template? Is the DNA template part of a larger molecule, such as a plasmid, or is the template its own fragment of DNA? Does the DNA template get excised from a plasmid using the CRISPR nuclease or another mechanism?

Templated editing can also be achieved without delivery of DNA template. Base editing enables a narrow range of sequence replacements without the need for a nucleotide template (Komor et al., 2016; Kantor et al., 2020). Prime editing allows the template to be delivered as RNA, typically in the form of an extension of the gRNA (Anzalone et al., 2019). Prime editing enables any substitution, precise deletions, and insertions up to 40 or even 80 nt using single or paired prime editors. Prime editors combined with an integrase enable much larger insertions, although these require the desired insert to be delivered as DNA which could possibly integrate in off-target locations (Anzalone et al., 2022; Yarnall et al., 2022). For a review of prime editors and base editors in plants, see (Xie et al., 2022; Azameti and Dauda, 2021).

To date, a wide range of gene editing reagents have been demonstrated using plasmid-based methods, but only a subset has been purified as ribonucleoproteins and demonstrated to function in plants. If considering ribonucleoprotein-based delivery, it is important to first establish whether the desired engineered nuclease, base editor, or prime editor is commercially available as protein or can be produced in-house. The scarcity of commercially available purified fusion proteins implies that the generation of transgene-free plants resulting from these approaches may depend on improvements of DNA-based delivery.

##### 2.1.1.5 Cargo size

Several of the delivery vehicles described below have limited cargo capacity and may be unable to deliver an entire SpCas9 protein, mRNA, or DNA. This limitation is very familiar to researchers in the medical and mammalian fields, who are most concerned about size limitations to AAV-based delivery, thus much progress has been made on this topic recently. For context, the coding sequence of LbCas12a is >3.5 kb, SpCas9 is >4 kb, and the first published prime editors and base editors are typically >6 kb, in each case excluding any promoter or other expression element. Several small nucleases have been published with coding sequences as small as ∼1.5 kb. Full elaboration on the recent advances in small nucleases is beyond the scope of this review, however, the following reviews and publications may be helpful (Wu et al., 2021; Wang et al., 2022a; Wang et al., 2023; Al-Shayeb et al., 2022; Goltsman et al., 2022; Li et al., 2023; Liu et al., 2019a; Pausch et al., 2020).

#### 2.1.2 Choose the vehicle

Plant cell walls and cell membranes are a potent barrier against the entry of foreign DNA, RNA, or protein, and these barriers must be overcome by force or cunning without overly damaging the cell. The diameter of gene editing reagents is ∼10 nm (RNPs), ∼25 nm (condensed plasmid), and up to ∼100 nm (uncoiled plasmid), while the plant cell wall has a size exclusion limit of approximately 10 nm (Wang et al., 2021). For a review of delivery methods for plants, see (Ghogare et al., 2021; Bhattacharjee et al., 2023). For a recent succinct review on CRISPR delivery mechanisms to plants, including transgene-free methods, see (Laforest and Nadakuduti, 2022). For information about CRISPR reagent delivery and the applications of morphogenic genes to this process, see (Gordon-Kamm, 2021; Che et al., 2022; Yarra and Krysan, 2023; Debernardi et al., 2020). Some delivery methods can be adapted to a tissue-culture-free protocol (see Supplementary Note S3). The primary tools to deliver reagents into plants are *Agrobacterium* and particle bombardment (gene gun), with other tools such as protoplast transfection, microspore transformation, nanotubes, silicon carbide whiskers, microinjection, pollen-based HI-Edit, and viral delivery used to varying degrees based on the crop and the application.

##### 2.1.2.1 Agrobacterium as a vehicle

Today, *Agrobacterium*-mediated delivery of T-DNA is widely used in many species to achieve consistent, low-copy transgene insertions and to deliver GE reagents encoded in transgenes. These methods take advantage of a pathogen’s ability to bypass the cell wall. Since this method’s beginnings in the 1970s, various protocol improvements have expanded the host range of *Agrobacterium*: tissue pre-treatments, culture with acetosyringone (reviewed in (Gelvin, 2003; Kausch et al., 2021)), use of supervirulent plasmids (Anand, 2018), assembly of very large T-DNAs directly in *Agrobacterium* (McCue et al., 2019), use of species other than *A. tumefaciens* (Cao et al., 2023), blocking host defense factors using the *Pseudomonas* Type III secretion system to deliver effectors (Raman et al., 2022), and using a variety of morphogens to improve regeneration of transformed tissue (Lowe et al., 2016; Gordon-Kamm et al., 2019; Hoerster et al., 2020; Debernardi et al., 2020; Che et al., 2022).

*Agrobacterium* delivers a single stranded DNA, called a T-DNA, excised from a plasmid and defined by Left Border and Right Border sequences. The mechanism uses several virulence proteins, in particular VirD2 and VirE to excise the T-DNA from the Ti plasmid, protect its ends and length, and pull it into the plant cell’s nucleus—VirD2 has a strong nuclear localization sequence. The multiple components of gene editing, such as the nuclease, guide RNAs, and repair template(s) are typically cloned into a single binary plasmid, along with any visual or selectable marker(s). The resulting plasmids can be quite large. If the relative amounts of any components should be optimized, this must usually be done through choice of expression elements, as the T-DNA is transferred as a single unit. T-DNAs ranging from 5 to 30 kb are used routinely, but larger T-DNAs, especially those in excess of 100 kb are transferred only at very low efficiency, reviewed in (Xi et al., 2018). DNA with synthetic chemical modifications, which might be desirable for a homology-directed repair template, cannot be introduced and maintained in the T-DNA region of the plasmid from which *Agrobacterium* transfers DNA into a plant cell.

Recently, modified strains of *Agrobacterium* with a *Pseudomonas* type III secretion system (PT3SS) have been demonstrated to deliver the protein AvrPto and fragments of GFP (Raman et al., 2022), reminiscent of how TALEs are delivered by the type III secretion system from *Xanthomonas* (Bogdanove, Schornack, and Lahaye, 2010). While particle bombardment (described below) can deliver ribonucleotide complexes, it is not clear whether the modified *Agrobacterium* would also deliver RNP complexes, or would need to deliver the protein and RNA components separately.

The main advantages of *Agrobacterium* as a vehicle are its ability to transfer an easily customized piece of DNA (and possibly a customized small protein) into a plant cell and that *Agrobacterium* protocols have been used and improved upon for decades. Undeniably, *Agrobacterium* is a powerful tool for low-copy integration of transgenes. The main limitations of *Agrobacterium* as a vehicle are the limited host range (many cultivars and species remain recalcitrant to *Agro*-transformation), requirement to clone relatively large binary plasmids, and that protocols are generally optimized for low-copy integration of transgenes, not for transgene-free gene editing outcomes.

##### 2.1.2.2 Particle bombardment (gene gun) as a vehicle

Particle bombardment uses force to bypass the cell wall, which, while crude, means this method works for almost any species and cultivar. In addition, particle bombardment is a flexible technique where the desired protein, RNA, or DNA (circular or linear, double- or single-stranded) is randomly and non-covalently attached to millions of microscopic gold (or tungsten) microcarriers (0.6 μm and 1.0 μm diameter particles are common). Reagents are typically applied to microcarriers in an aqueous solution and precipitated using spermidine/CaCl_2_/PEG, glycogen, or a cationic lipid reagent (e.g., TransIT-2020) (Ratnayaka and Oard, 1995; Svitashev et al., 2016; Imai et al., 2020; Miller et al., 2021; Dong et al., 2021). The particles provide mass and are accelerated with pressurized helium in a gene gun, thus providing sufficient force to overcome the cell wall. A very small fraction of these particles results in successful delivery of reagents to a plant cell which survives. The reagents must detach from the particle in the target cell compartment, often the nucleus, or be transported into it.

A frequently cited downside to particle bombardment is that the process damages DNA, causes occasional chromosome rearrangements (Yue et al., 2022), and creates DNA breaks into which the reagent DNA (or carrier DNA) may integrate randomly (Liu et al., 2019b). In addition, the loading of DNA onto particles may be uneven (i.e., many particles contain no DNA, while others contain many), and high copy tandem insertions of transgenes are not uncommon (Poddar et al., 2023). These disadvantages, combined with improvements in *Agro*-transformation techniques, led to a decrease in the popularity of particle bombardment for purposes of making transgenic plants.

A key reason for the revival of particle bombardment is that gold microcarriers can deliver protein as well as nucleic acids (Martin-Ortigosa and Wang, 2014; Miller et al., 2021). The particle bombardment protocol must be modified to prevent denaturing the gene editor RNPs, which can tolerate desiccation, but not suspension in pure ethanol (Svitashev et al., 2016). Several protocols for delivery of CRISPR RNPs have been published by both academic and industry groups (Svitashev et al., 2016; Liang et al., 2017; Poddar et al., 2023). While these protocols are reproducible, delivery of proteins (and ribonucleoproteins) by particle bombardment has not been optimized to the same extent as delivery of DNA (Ratnayaka and Oard, 1995; Martin-Ortigosa and Wang, 2014; Miller et al., 2021). For example, “no studies to our knowledge have been carried out to quantify the amount of Cas9-RNP that is truly adsorbed to gold particles by these methods. Such data would be valuable to avoid underloading or overloading gold particle preparations with Cas9-RNPs.” (Poddar et al., 2023). While studies from mammalian tissue culture indicate that transfected RNPs are degraded within <48 h of delivery, one recent report suggests that traces of RNPs delivered to plant cells by bombardment remain detectable, and possibly even active, for as long as 14 days (Poddar et al., 2023).

Another major advantage of particle bombardment is that this method works for a much wider range of species, cultivars, and explants, because it relies on force rather than a “clever” pathogen. Bombardment is typically used with immature embryos, callus, or explants which are regenerated through callus. In addition, *in planta* particle bombardment, targeting the embryonic shoot apical meristem from dissected imbibed wheat seeds, was published as an alternative, and used to deliver transgenes encoding CRISPR reagents (Hamada et al., 2017; Imai et al., 2020). Presumably a similar approach is possible for delivering RNPs in a DNA-free way, since the published approach does not rely on selection using transgenes.

The main advantages of particle bombardment as a vehicle are the ability to deliver anything that can be coated onto microcarriers and the ability to reach an exceptionally wide range of explants, cultivars, and species. The main limitations of particle bombardment as a vehicle are due to the imprecision of loading of reagents onto microcarriers and potential for damaging the genome through force.

##### 2.1.2.3 Viral vectors as vehicles

Instead of bypassing the cell wall by force or using *Agrobacterium* to deliver DNA, several teams have borrowed another plant pathogen—viruses—to bypass the cell wall in a method called virus-induced gene editing (VIGE). This approach is analogous to AAV-mediated delivery to mammalian cells. The requirements for viral delivery of gene editing reagents to plant cells are host range and being able to fit the gene editing cargo into the genome of the virus. In some cases, the virus can fit a gRNA, but the nuclease must be expressed in the plant as a transgene, meaning that those methods are not exactly relevant to this review. For a recent succinct and illustrated review of viral delivery methods, see (Laforest and Nadakuduti, 2022). The benefits of viral vector delivery of editing reagents are two-fold. First is the possibility to produce heritable edits without the need for tissue culture, such as in (Li et al., 2021), and second is the ability to produce edited plants which do not contain the DNA encoding the editing components in their genomes.

Briefly, the approach involves delivery of the viral genome on a T-DNA vector via *Agrobacterium* infiltration of leaves on an intact plant. The T-DNA can integrate locally at the site of infiltration, but when expressed from the T-DNA, the viral genome containing CRISPR reagents can form viral particles and move systemically through the plant. Editing can thus theoretically occur from the expressed viral genome in many plant cells, which do not contain the DNA encoding the editing reagents. If meristematic cells are also “infected” with the mobile viral genome, editing in these cells can be propagated to embryos, thus resulting in a transgene-free edited plant obtained without the need for tissue culture. Alternatively virus-encoding *Agrobacterium* can be infiltrated into an intermediate host, such as *Nicotiana benthamiana*, to produce viral particles, which can be purified and applied to the target plants.

Though promising, this approach has five main challenges. The first challenge is the limitation on the size of a modification which can be tolerated within a typical plant viral genome. In most cases reported to date, the cargo capacity of compatible plant viral genomes is ∼1 kb, while the size of a typical Cas protein is ∼4 kb, and that of a Cas9 plant expression cassette is >6 kb. Thus, most studies have focused on viral delivery of gRNAs to a transgenic plant expressing a Cas9 protein. This approach is promising for species for which the elimination of tissue culture is the major benefit of this system, since Cas9 can be crossed out in subsequent generations. However, for species with vegetative propagation, this approach presents a limitation. At least one study reports successful delivery of an entire CRISPR-Cas cassette to tetraploid tobacco using a plant virus belonging to the Rhabdovirus family of negative strand RNA viruses with a larger cargo capacity (Ma et al., 2020). Additionally, in this case, the virus could be delivered directly to the plant without the need for an *Agrobacterium* vehicle. Despite this success, the challenge of engineering novel plant viral vectors such as this can limit the adoption of this approach. Additionally, due to host-range limitations of most viruses, additional viral genome engineering may be required to expand the range of species which could benefit from this approach, further deepening the technical challenge of its use. Another approach to overcoming the cargo size limitation is the use of distinct or satellite viruses (Maher et al., 2020). A promising approach to overcome the cargo size limitation of more commonly used viral genomes is to use tiny Cas proteins so that the entire CRISPR-Cas cassette would “fit” within 1 kb. Multiple Cas proteins less than one quarter the size of typical Cas9/Cas12a proteins have now been described (see previous section on cargo size). The approach of combining tiny Cas proteins with the viral delivery approach is promising for delivery both tissue culture-free and transgene-free benefits of the system to vegetatively propagated species.

A second challenge to the viral delivery of editing components is the extent of systemic movement, which directly translates into the efficiency of obtaining the desired edited plants. One successful approach to increase the cell to cell mobility of sgRNAs has been the addition of short sequences in the 3′ of Cas9 gRNAs such as tRNA motifs and FLOWERING LOCUS T (FT)-like motifs (Ellison et al., 2020; Li et al., 2021).

The third limitation is the fact that often the choice of an efficient viral vector is specific to a particular group of plants (often monocots vs. dicots), requiring the testing and optimization of viral vectors directly in the crop species or its relatives. For example, although some of the foundational research for viral delivery was done in *Nicotiana* species using either positive strand DNA Geminiviral or positive strand RNA viruses like tobacco rattle virus, for grass crops such as maize and wheat, foxtail mosaic virus (+RNA) and barley stripe mosaic virus (-RNA) have been used. For example, foxtail mosaic virus has been used to deliver CRISPR reagents to maize and *Setaria* (Mei, Beernink, et al., 2019) and sugarcane mosaic virus, which can tolerate insertions of at least 1809 nucleotides has been used to deliver GFP, Gus, and Bar into maize (Liu, et al., 2019). Recently TSWV, a virus with broad host range in dicots and even a few monocots (Cha and Mau, 1987) was reported to deliver full-sized Cas9 reagents (Liu et al., 2023).

Fourth, because of the non-targeted nature of systemic viral movement, obtaining a non-chimeric plant necessitates passage through a single cell bottle neck such as in seed formation, making this a challenging approach for vegetatively propagated crops, complex hybrids or crops with long generation times.

Finally, fifth, the absence of virus or viral genome from the resulting plants is crucial from a crop product perspective. Many viruses are excluded from the germline (single-cell-bottleneck), largely alleviating this concern. In some cases, the virus can be modified to be non-insect-transmissible and curable (Liu et al., 2023). In other cases, antivirals can be used to cure the viral infection after editing, although this may not be especially challenging in vegetatively propagated crops (Voytas, 2023).

The main advantages of viral vectors as a vehicle are the potential for transgene-free, and perhaps also tissue-culture free delivery of gene editing reagents. The main limitations of viral vectors as a vehicle are an especially narrow host range for each particular virus and a cargo size capacity typically too small to contain the entire expression cassette for gRNA(s) and a nuclease.

##### 2.1.2.4 Nanomaterial-mediated delivery vehicles and other physical methods

Many methods besides the gene gun and pathogen-based vectors have been tested, but to date none are widely adopted for use with intact plant cells. When the cell wall is removed to form a plant protoplast, the delivery vehicle only needs to bypass the cell membrane. In plant protoplasts and mammalian cell culture, lipofection, lipid nanoparticles (LNPs), electroporation, and PEG/Ca^2+^-mediated transfection can be used to deliver proteins, RNA, or DNA (Woo et al., 2015; Murovec et al., 2018; Andersson et al., 2018; Furuhata et al., 2019; Wu et al., 2020; Wang et al., 2020; Badhan et al., 2021). In the species where protoplast regeneration is practical, such methods may offer a convenient and extremely efficient method for transgene-free gene editing. Unfortunately, in most species, protoplast regeneration is not straightforward. An alternative explant that may be able to bypass the plant cell wall is a zygote shortly after gamete fusion, which can be transfected using similar methods as protoplasts (Toda et al., 2019). However, researchers are often limited to working on explants with mature cell walls.

Various nanomaterial-mediated delivery options have been published to overcome the plant cell wall. Nanoparticles offer entry into plant cells without the use of crude force (unlike particle bombardment) and thus hold the theoretical ability to provide tissue culture free delivery of reagents. However, reagent delivery is still limiting due to the custom chemistries required to reversibly bind different reagents to the nanoparticles. Single wall carbon nanotubes can deliver transient expression of plasmids up to ∼10 kb, including gene editing reagents, and siRNA (Zhang et al., 2022; Demirer et al., 2020, 2019). For a review of nanotube and nanoparticle methods, see (Demirer et al., 2021). Clay nanoparticles can deliver RNA into intact plant cells (Yong et al., 2022, 2021). While not peer-reviewed, carbon nanodots have been reported to deliver plasmid DNA to intact plant cells (wheat leaves) which expressed fluorophores and demonstrated editing. (Doyle et al., 2019). DNA nanostructures have been reported to deliver siRNAs and may be adaptable to delivering gene editing reagents (Zhang et al., 2019a). Another recent report describes transformation of maize pollen using DNA-coated magnetic nanoparticles (Wang et al., 2022b). Nanodelivery of RNPs has not yet been demonstrated in intact plant cells, but has been shown in mammalian cells (Wang et al., 2016; Mout et al., 2017; Mout et al., 2017; Lee et al., 2017; Wei et al., 2020). For a review on protein delivery to intact plant cells, see (Wang et al., 2021). For reviews of nanocarrier delivery methods including carbon nanotubes, carbon dots, and mesosporous silicon nanoparticles, see (Kumari and Pratap Singh, 2021; Mujtaba et al., 2021), and for a review focused on gene editing, see (Ahmar et al., 2021).

Besides nanomaterials, cell penetrating peptides, which employ endosomal entry and escape mechanisms, have been demonstrated to deliver DNA and proteins into plant cells after infiltration into leaves in dicot plants (Ng et al., 2016; Chuah and Numata, 2018; Terada et al., 2020). Combinations of cell penetrating peptides have recently been reported to enable delivery of reagents to intact plant cells, possibly through a mechanism similar to micropinocytosis (Miyamoto et al., 2022). For a review of peptide-based and nano-based delivery methods for gene editing into intact plant cells, see the review (Arya et al., 2021).

The advantages and limitations of these delivery methods are difficult to generalize and their adoption is not yet widespread.

##### 2.1.2.5 Grafting and other biological delivery methods

The two delivery methods in this section take inspiration from the ways in which DNA, RNA, and proteins move into intact plant cells using mechanisms found in plant biology. Both of the following methods also have the advantage of being tissue-culture-free.

The natural process of fertilization requires genetic material from two cells to fuse. The delivery method called HI-EDIT (haploid-induction editing) takes advantage of pollen as a vehicle for gene editing reagents (Kelliher et al., 2019). First, a transgenic line is made which contains the gene editing reagents and a haploid inducer genotype. This line is used as the pollen donor. The target variety is pollinated by this pollen donor, receives and expresses the gene editing reagents, and editing can take place in the maternal genome. However, fusion of genomes from the pollen and from the egg does not occur. Instead, the genome from the pollen is rejected very early, along with the gene editing reagent transgenes, and the egg develops into a haploid zygote. This creates a short window when the editing reagents can be expressed and produce an edit. The haploid genome can be doubled to produce a homozygous edited, transgene-free plant without tissue culture. Note that this method is entirely different from delivering DNA to pollen, as in (Wang et al., 2022b).

Plant tissues communicate using a variety of small molecules, but also mobile RNAs. Recently, a method was published to work in several dicot species which takes advantage of this inherent biology to move gRNA and mRNA encoding gene editing reagents into the target plants. The method relies on grafting wild-type target scions onto transgenic rootstocks which deliver mobile mRNA and gRNA (Yang et al., 2023). This method produces mosaic plants, which must pass through a single cell bottleneck such as meiosis to produce the next-generation where the edits become fixed.

The main advantage of these methods is the potential for simultaneously tissue-culture-free and transgene-free gene editing and in enabling editing of extremely transformation-recalcitrant cultivars since the donor plant can be made in an easily transformable cultivar. The main limitations of both methods are that transgenic reagent donor plants must be generated, the haploid induction and/or grafting techniques are not possible in all species or cultivars, and the process is not very efficient even in the model where it was developed.

### 2.2 Step 2: recover edited plant

Recovery of edited plants is also a challenge, even after efficient delivery of reagents is achieved. Once delivered, CRISPR reagents are generally very efficient at editing DNA, but plant regeneration from edited cells is limiting and time-consuming. Even the most efficient delivery methods described above do not reach 100% of the target cells, meaning plants regenerate from a mixture of edited and unedited cells. Adding difficulty to this process, the delivery method may slow the growth of edited cells, which may lead to faster growth of the un-edited cells. For these reasons, recovering an edited plant is an added challenge in the context of transgene-free methods where researchers are limited in the use of traditional transgenic antibiotic or herbicide selection to increase the efficiency of the process.

Recovering edited plants can be made more efficient using selection to reduce the division and growth of cells which did not receive reagents. The number of plants which must be screened for the desired edit can be reduced by many orders of magnitude by using a chemical (antibiotic, herbicide, etc.) selection scheme to kill cells which received no reagents. This approach places less pressure on efficient delivery because it can enrich for the desired plants.

Historically, selectable markers have been transgenes, which are undesirable in the final product. In the next two sections, we describe approaches used to employ selection with transgenes, yet without leaving those transgenes in the final product. In the third section, we describe methods which use selection without ever using a transgene. In the final section we describe methods with no selection, which rely on very efficient reagent delivery and/or very large sample sizes.

#### 2.2.1 Full transgenic selection followed by transgene removal

This method still relies on transgene integration and standard transgenic selection. As such, it is only quasi-transgene-free and open to interpretation whether it fits the definition of transgene-free gene editing. In many crops, transgene-free edited lines can be produced using classical breeding. This is a very simple approach to generating transgene-free edited plants in species where performing such crosses is routine. However, in species with long generation times, which are propagated vegetatively or through apomixis, and/or in complex hybrids, such crosses may be impractical or impossible.

Even in cases where breeding away a transgene is feasible, several tactics using visual markers or lethal genes (e.g., the Transgene killer CRISPR system) have been developed to make the process more efficient, reviewed in (He and Zhao, 2020). One can reduce the labor in screening away transgenes by using a visual marker to quickly identify seeds with a transgene or by using a conditionally-lethal gene to eliminate transgenic seeds or seedlings (He et al., 2018; Aliaga-Franco et al., 2019; He et al., 2021). For a review on the application of these approaches for screening away transgenic plants see (Yau and Stewart, 2013) and (Breyer et al., 2014).

An alternative to segregating away transgenes through breeding is to remove the transgenes within the same generation. For example, the material is transformed with a single T-DNA containing transgenes for all required editing reagents and antibiotic resistance transgenes. In addition, the T-DNA contains sequences which can trigger transgene excision (Wang et al., 2023a). Laforest and Nadakuduti (2022) provide a recent review into these methods.

One such method uses the same CRISPR nuclease to create a cut at two gRNA locations within the transgene. The gRNAs could be the same ones that are being used to edit the genome at the locus of interest. When the CRISPR nuclease makes a double-strand break at both gRNAs simultaneously, the linear piece of DNA containing all the transgenes can “drop out” of the genome. The genome retains only a gRNA “footprint,” and if *Agrobacterium* was used, also portions of the LB and RB (He et al., 2021).

A second method includes an additional Cre-recombinase transgene, usually with an inducible promoter, and LoxP sites flanking the region to be excised. When the two LoxP sites face the same direction, cre-recombinase activity will result in the formation and eventual degradation of a loop of DNA. The genome retains only a LoxP “footprint” and portions of the LB and RB. Alternatively, excision with a piggyBac transposase or other systems may enable removal without a residual footprint (Nishizawa-Yokoi et al., 2014, 2015).

In either design, plantlets can be selected using typical transgenic selection schemes and T0 plantlets with single-copy transgene insertions and homozygous edits are chosen. The transgenes are excised within the T0 plantlets, and the resulting plantlets have homozygous edits and only “footprints” of the transgenes in random genome locations. The excision could happen sequentially, possibly resulting in a mosaic plant where some of the cells contain intact transgenes while other cells contain only the resulting “footprints.” Adjustments to the tissue culture may be necessary to regenerate non-mosaic, fully-excised plants (He et al., 2021).

Full transgenic selection remains a useful option for gene editing when the final product will be transgenic, such as when using GE technology for precise transgene insertion (i.e., site-directed integration). For example, site-directed integration can be achieved with FLP-FRT recombination (Anand et al., 2019). For a review of site-directed integration, see (Dong and Ronald, 2021). Inserting into a known location reduces the burden of finding the location of a randomly integrated transgene, reduces event-to-event variability, and possibly streamlines the regulatory process. Favorable locations in the genome, which offer high gene expression and do not disrupt genes, regulatory elements, or transposons are called “genomic safe harbors” or colloquially known as “landing pads.” Furthermore, the chosen location may be favorable for trait introgression, trait stacking, or trait pyramiding. The inserted transgenes can contain only trait genes, or can also contain widely used and de-regulated transgenes (e.g., nptII). In a few convenient cases, trait genes can be selectable by chemical means or visually. For example, the carotenoid biosynthesis pathway offers a visual phenotype, which was used to demonstrate the transgene insertion into a genomic safe harbor (Dong et al., 2020).

If the selectable marker gene is in the same vector as the genes encoding CRISPR gene editing reagents, these will almost always integrate together. The CRISPR reagents will continue to be expressed and continue editing—on-target until all copies of the target are edited, and possibly also off-target (Gong et al., 2021). If the genes encoding CRISPR reagents are in separate T-DNAs or separate plasmids, it is possible that only the selectable marker genes integrate, and the resulting plant is transgenic but without continued editing. If the selectable marker genes are delivered as DNA and the CRISPR reagents are delivered as mRNAs or ribonucleoproteins, then the most likely outcome is an integrated selectable marker gene and no continued editing. If the recovered plant “escapes” selection after editing occurs, then it would be exactly the kind of edited, transgene-free plant desired. This approach is described further in the next section.

#### 2.2.2 Transient expression for transgene selection to avoid transgene integration

In most cases, the desired trait is not easily selectable, and one must still rely on a transgene for selection. All hope is not lost for recovering transgene-free plants, however, because the selectable marker may be expressed “transiently” (He et al., 2021). The gene editing reagents can also be expressed transiently, produce an edit, and never integrate (Gelvin, 2022). This has been demonstrated to be possible from T-DNA delivered by both wild-type *Agrobacterium* (Veillet et al., 2019; Danilo et al., 2019; Bánfalvi et al., 2020; Chen et al., 2018; Zhang et al., 2023) and *Agrobacterium* mutants to enhance transient expression and/or reduce integration (Mysore et al., 1998; Gelvin, 2003; Lee, 2022). After using ordinary *Agrobacterium*, 3 days of kanamycin-based selection was sufficient to enrich for edited potatoes without transgene integration (Bánfalvi et al., 2020). Some groups have used a combination of “transient transgene” selection combined with an herbicide-based selection for an edit in an herbicide target gene. For example, one group used kanamycin selection for a KanR transgene initially after transformation, then switched to chlorsulfuron to select for ALS edits, although the relative contributions of kanamycin and chlorsulfuron are unclear (Veillet et al., 2019).

Because integration into the genome is generally understood to be a one-way process, many of the DNA-based approaches described above rely on expression from extra-chromosomal DNA, without integration. For these applications, it may be beneficial to incorporate a component to discourage integration and/or to select against cells in which integration occurred. These are similar to the tactics for identifying non-transgenic plants described in the above section on full transgenic selection, such as barnase, slower growth of RUBY transgenic plants, and conditional toxicity of certain compounds when combined with a transgene; for example, the CodA transgene converts nontoxic 5-FOA into toxic 5-FU (Koprek et al., 1999; Breyer et al., 2014; Oliveira et al., 2015; He et al., 2018; Aliaga-Franco et al., 2019; Bánfalvi et al., 2020; He and Zhao, 2020; He et al., 2021; Kumar et al., 2022). Such protocols offer a path to removing transgenic plants by selection against transgenes instead of laborious and costly screening by molecular methods.

The main advantage of this approach is that well-established protocols for transformation (e.g., delivery of DNA by *Agro*-transformation or gene gun) can be used. The main limitations of relying on incomplete selection following the delivery of transgenes is low efficiency and few reports to date, the need to optimize the marker, dose, and duration of selection for each explant, as well as that screening still needs to be applied to verify that the edited plants lack transgenes (Chen et al., 2018).

#### 2.2.3 Non-transgenic selection

In a few lucky instances, the desired edit produces a phenotype which is visible or selectable early on in an experiment. In these instances, the edit itself can be a transgene-free visual marker.

While not useful for commercial products, the knockout (loss-of-function) of the *PDS* gene to produce an albino plant is a well-established model, quite useful for proof-of-technology experiments. Use of the albino phenotype increased the editing rate in tobacco in an otherwise selection-free experiment from approximately 2.5% to approximately 50% (Chen et al., 2018). Examples like these albino plants and the carotenoid-expressing plants in the above section still required visual screening to identify the edited or modified plants.

Selection with a chemical is less labor intensive than visual screening. In typical transgene-based selection, a chemical such as kanamycin is used to kill tissue without the kanamycin resistance transgene. In an edit-based approach, a chemical, typically an herbicide, is used to kill un-edited tissue, while an edit provides herbicide tolerance (Shillito et al., 2021). Certain forms of herbicide tolerance require a particular amino acid substitution, making them more adaptable to edit-based selection. For example, EPSPS substitutions confer glyphosate tolerance in cassava (Hummel et al., 2018), rice (Jiang et al., 2022), and other crops. Similarly, particular amino acid substitutions in ALS confer tolerance to several herbicides including chlorsulfuron and imidazole (Yu et al., 2010), and have been used to select for edited plants with herbicide tolerance in numerous species (Jiang et al., 2020; Butt et al., 2020; Zhang et al., 2019b; Malabarba et al., 2020; Huang et al., 2022; Alquézar et al., 2022; Oz et al., 2021). If edits in a new herbicide target are the goal of an experiment, selecting directly with that herbicide is logical. For example, Xu et al. (2021) used saturation mutagenesis with prime editing to screen for new substitutions in OsACC1, an herbicide target.

In these examples, the challenge is that a very precise amino acid substitution is required, and it is not clear if imprecise knockout edits in the other or additional gene copies are tolerated. However, since the desired result is likely to be a phenotype of herbicide tolerance, selection with the target phenotype is a bonus. However, some researchers have noted that selection with herbicide *during tissue culture* has not enriched for edited herbicide-tolerant plants (Oz et al., 2021).

The challenge with most herbicide tolerance models is that precise templated editing is typically required. Because homology-directed repair is so far inefficient in plants and templated editing methods are typically more challenging than knockout methods, it may be desirable to develop a knockout-edit-based marker to enable research. A few knockout edits have visible phenotypes in plants—such as knockout of the *PDS* gene producing visibly albino leaves and knockout of nitrate reductase allowing growth on chlorate. However, these have the disadvantage of being deleterious, and removing such a marker edit is equally challenging as removing a marker transgene. For this reason, a convenient marker gene’s knockout phenotype should be neutral under field conditions and only apparent under laboratory conditions (e.g., upon exposure to a chemical). For example, knockout of the PtUMPS (URA3 homolog) in diatoms makes the organism tolerant to 5-FOA, and knockout of PtAPT (an adenine phosphoribosyl transferase) makes the organism tolerant to 2-FA (Serif et al., 2018).

Several groups have demonstrated herbicide-based selection for an edit in ALS resulting in a transgene-free plant. In most cases the reagents were delivered as DNA through *Agro*-transformation, where editing takes place due to transient expression from T-DNA (Veillet et al., 2019). Since a T-DNA is present, sometimes the herbicide-based selection was combined with a transient transgene selection approach (as described in the above section). Some studies have also demonstrated multiplex editing of an herbicide gene and a non-herbicide gene in the same plant, although traditional transgene-requiring selection was also used in these experiments (Shimatani et al., 2017, 2018; Li et al., 2022).

Since transgene-free editing following herbicide-based selection as well as multiplex gene editing have both been demonstrated, several researchers have proposed a co-editing approach to achieve transgene-free gene editing (Veillet et al., 2019; Huang et al., 2022), similar to that used in non-plant systems (Arribere et al., 2014). One example of a transgene-free co-editing strategy with herbicide selection for an edit was very recently published as a pre-print (Huang et al., 2023). The researchers used a base editor targeting ALS to achieve resistance to the herbicide chlorsulfuron combined with editing by Cas12a for the trait(s). *Agro*-transformation was used to deliver these reagents in a T-DNA along with a GFP transgene used to aid in screening away plants with transgene integration. In tobacco, tomato, potato, and citrus, up to half of the herbicide tolerant plants recovered had biallelic or homozygous editing at the target gene.

The main advantage of selecting for an edit (or a co-edit) is that it allows for a possibly more efficient protocol where un-edited plants are removed by chemical selection. The main limitations of a co-editing approach are that chemical or herbicide selection need to be developed in a way that targets a native gene, typically requiring templated editing, and that the sequence change of the co-edit may be undesirable.

#### 2.2.4 No selection (“Brute force”)

The ideal method in medicine and agriculture is delivery so efficient that selection is unnecessary. A method to deliver the exact sequence change required for a trait without adding or changing additional regions is highly desirable for both transgenics and in gene editing. Unfortunately, this approach usually results in very few homozygous edited and non-mosaic plants, a few heterozygous or mosaic edited plants, and a vast quantity of wild-type plants.

One way to increase the efficiency of edited plant recovery is to vastly increase the efficiency of delivery. One can choose explant types without cell walls and simply deliver enough reagents that a very large fraction of the cells are edited. In plant protoplasts, reagents can be delivered to the majority of cells (Lin et al., 2018), and in some species regeneration from protoplasts is feasible and an extremely efficient way to recover transgene-free gene edited plants (Andersson et al., 2018; Murovec et al., 2018; Choi et al., 2021; Tricoli, 2022). In most species, however, regeneration of healthy plants from protoplasts is not feasible. Nevertheless, protoplasts provide a convenient method for rapid screening of reagents in plant cells (Lin et al., 2018) (see Supplementary Note S4).

Multicellular tissues with intact cell walls are orders of magnitude more challenging for reagent delivery. Unfortunately, in almost all cases, an entirely selection-free method results in recovery of thousands of unmodified plants before a single edited plant is found. Without selection pressure to reduce the background, this method is labor-intensive and requires high-throughput screening. To make this method feasible and cost-effective, researchers typically employ methods such as pooling samples, and screening these pools using sensitive methods, such as amplicon sequencing by Illumina (Liu et al., 2019), high-resolution melt (Chen et al., 2018), fragment analysis, or digital PCR (Peng et al., 2020), reviewed in (Shillito et al., 2021). A few exceptional successes (>3% efficiency) have been reported in wheat and maize using entirely no selection following particle bombardment with ribonucleoproteins (Dong et al., 2021; Poddar et al., 2023; Liang et al., 2017). Likely the efficiency is lower as unsuccessful experiments are under-represented in literature; efficiencies well below 1% are not uncommonly heard at conferences.

The main advantage of this approach is that it is conceptually simple and entirely free of exogenous DNA. Unfortunately, given the limitations of current delivery methods in plants, this approach is typically extremely inefficient (Tsanova et al., 2021).

## 3 Summary and conclusion

When gene editing was adopted in crops, the GE techniques were developed to fit well into pipelines built for breeding with inbred parents, hybrid seeds, and transgenic trait introgression. Adoption of new GE technology in crops like maize typically involves *Agro*-transformation of GE transgenes into a parent line, allowing editing to occur, breeding away the transgenes, and introgression of the edited trait to make elite hybrid seeds. This approach has not been practical for crops that are recalcitrant to transformation, have long generation times, are vegetatively propagated, or require complex heterozygosity for elite varieties. This has driven demand for protocols to recover transgene-free edited plants without requiring breeding.

The current state of the field is a rapidly evolving mixture of solutions, each built for a niche application. For example, pollen-based delivery methods like HI-EDIT offer a transgene-free solution for crops with short generation times which already employ doubled haploid technology (Kelliher et al., 2019). Transfection of protoplasts with ribonucleoproteins offers an efficient route to transgene-free editing for crops with efficient protoplast regeneration protocols. Grafting of target variety scion onto rootstock which can deliver GE reagents as mobile RNAs appears to be a promising new way for some dicot species, assuming the efficiency of recovering non-mosaic edited plants is improved. Virus-based delivery is an overall promising method since it offers a tissue-culture free method in addition to transgene-free, but the hurdles of host range and cargo size limitation remain.

Overall, the field has seen advances in all the components: cargo options, vehicle designs, and plant recovery approaches. Because of the constantly changing landscape, we cannot pick a single, superior method, and it is difficult to predict which approach will be the next big thing (Tsanova et al., 2021). There is not an obvious candidate method which we could predict will become dominant in the short-term. More likely, several methods will expand to solve challenges in a crop-specific manner. Perhaps there will eventually be a single perfect protocol to deliver and recover transgene-free edited plants in any species. When that emerges, it is possible that even crops without the aforementioned limitations will switch to this protocol for convenience and efficiency.

## Supplementary material

Supplementary Note S1 Possible editing outcomes.

Supplementary Note S2 Calculation of editing efficiency.

Supplementary Note S3 Additional info on delivery vehicles and tissue-culture-free methods.

Supplementary Note S4 Protoplasts and Microspores.
