# Supplementary material for: Strategies for delivery of CRISPR/Cas-mediated genome editing to obtain edited plants directly without transgene integration
Source: Front Genome Ed. 2023 Jul 20;5:1209586. doi: 10.3389/fgeed.2023.1209586 (PMC10398581; doi:10.3389/fgeed.2023.1209586)
Supplement: Supplementary file 1 [file DataSheet1.pdf]

Supplementary Material

Strategies for delivery of CRISPR/Cas-mediated genome editing to obtain edited plants directly without transgene integration

Zuzana Kocsisova*, Viktoriya Coneva

*** Correspondence: Zuzana Kocsisova**
zuzana.kocsisova@ctc.com.br

1 Supplementary Data

1.1 Supplementary Note 1: Possible editing outcomes

To optimize a process, it is important to understand what a successful outcome looks like. In a simple case of a single piece of DNA or a homogeneous monoploid genome, one only needs to describe the outcome of a single molecule. If no nuclease binding occurred, a sample is **unedited**. A sample can also be **unedited** if a DNA was cleaved and the double strand break or nick was repaired perfectly. A **knockout** or **indel** is the typical result when a double strand break from a single gRNA is repaired imperfectly by non-homologous end joining (or TMEJ/MMEH/Alt-NHEJ) resulting in a small deletion (<100nt, typically only ~1nt for Cas9 and <10nt for Cas12a), insertion (typically 1nt, occasionally a fragment of delivered reagents), or substitution (typically 1nt, or as mediated by local microhomology. Such an edit typically leads to a frameshift and a “knockout” loss-of-function, although an in-frame edit (e.g. a 3 base pair deletion) may not remove gene function. A **perfect edit** is the exact substitution, insertion, or deletion based on the template or base editor delivered. An **imperfect edit** occurs when using a template and only a portion of the desired insertion/substitution takes place and/or where some of the homology region is repeated or deleted. An **imperfect edit** can also occur when repair with base editor occurs with **bystander** edits at additional nucleotides within the editing window. In the case of a diploid or polyploid genome, the outcomes become progressively more complex. For example, a knockout edit could be heterozygous (e.g. one gene copy unedited, the other with a 1bp deletion) or biallelic (e.g. one gene copy with a 1bp deletion, the other with a 4bp deletion). If a template was used, one may find **mixed on-target edits**, where one copy of the gene is edited perfectly and another is edited either imperfectly, with bystander edits, or with knockouts/indels. The previous descriptions only examine the target locus. When examining the rest of the genome, **off-target editing** can occur independently of the on-target editing.

Mixtures of tissue further complicate how editing is measured. Mixtures come in several forms. Mixtures may be found in a suspension culture of protoplasts or callus. Mixtures may also be found in a single plant, such as chimeras and mosaics. A **chimera** resulted from a fusion of genetically distinct cells into a single plant, such as when several distinct cells in a callus culture form a somatic embryo, which then forms a new plant. The new chimeric plant may be a chimera if only one of the original cells received the reagents. In contrast, a **mosaic** resulted from a genetic

change in a subset of cells within a plant, such as when one cell became transformed with a CRISPR cassette, that single cell grew into a population of transgenic cells, formed an embryo which formed a plant, but the gene editing occurred in only a subset of cells later. In practice it can be difficult to distinguish the origin (mosaic vs chimeric) in plants with a mixture of genotypes.

Most GE methods rely on the DNA repair mechanisms in plant cells to heal the double strand break or nicked DNA, since an unhealed double strand break would be lethal to the cell and useless to agriculture.

1.2 Supplementary Note 2: Calculation of editing efficiency

To optimize a process, it is necessary to be able to measure outcomes. The calculation of editing efficiency in regenerated tissue, plantlets, or pools of plantlets "has the potential to be confusing" (Poddar et al. 2023). The <u>simplest</u> case is when whole plantlets are categorized into WT vs Edited. In this case, one can calculate # edited plants / # total plants = **fraction edited plants**. However, even within one plant, editing is not always clearly binary. Even in a diploid, the sample could be WT, heterozygous, biallelic, or homozygous. When multiple orthologs of a gene are targeted, and/or in a polyploid genome, the situation becomes more complex. One solution often employed is to set a threshold based on either the detection limit or on a case-by-case basis to continue using the **fraction edited plants** calculation to summarize the editing efficiency for a group of samples. Another calculation can be performed on individual samples to determine the fraction of DNA that is edited. For example, amplicon sequencing can result in some # edited reads / # total reads = **fraction edited DNA** in that sample. Further complicating the calculations: the tissue could be chimeric or mosaic, tissue samples may be pooled to save resources, or may be mixtures, such a protoplasts. These mixtures of cells need to be considered in the interpretation of the **fraction edited DNA** value. Finally, it is important to note that editing fractions may change over time for a few reasons. Editing reagents may continue to be present in the tissue, increasing the fraction edited. Edited and un-edited tissue may divide and grow at a different rate, changing the fraction edited. Also "only a subset of the originally edited tissue ultimately gives rise to an edited plant" (Poddar et al. 2023).

1.3 Supplementary Note 3: Additional info on delivery vehicles and tissue-culture-free methods

Several delivery methods that stopped being used for transgene integration are having a comeback in the era of gene editing. For example, use of the gene gun declined once consistent protocols were developed to generate single-copy transgenic events using *Agrobacterium* in a wide range of species and varieties. The vast majority of transgenic work now employs T-DNA delivery by *Agrobacterium* rather than the gene gun. The possibility of delivering gene editing reagents in a

DNA-free way, such as with ribonucleoprotein revived interest in the gene gun. It is possible that other methods which were attempted and discarded for transgenics may be found to be effective for delivery of gene editing reagents.

Gene editing has brought a resurgence in plant transformation methods, as gene editing reagents have distinct requirements compared to “simple” transgene integration. The field is no longer dominated by *Agro*-transformation for T-DNA delivery and low-copy “quality” events. In gene editing delivery by mRNA and by ribonucleoprotein is replacing DNA-based delivery, mainly driven by regulatory considerations of transgenes and an expectation of lower off-target editing. Both in gene editing and in transgenics, there is growing demand for **tissue-culture free** delivery methods. For gene editing, tissue-culture free has recently been demonstrated via viral or grafting-based delivery of cell-to-cell mobile reagents, and we look forward to these techniques reaching more and more crops. In a trend mirroring the medical field, the availability of smaller gene editors is enabling some of these delivery methods including viral delivery.

Agrobacterium-based delivery may also be desirable due to its potential for tissue-culture-free delivery. For decades, *Arabidopsis* has been transformed routinely using floral dip methods (Clough and Bent 1998). More recently, *Agro*-transformation with *Agrobacterium rhizogenes* was shown to be possible without tissue culture using a root Cut-Dip-Budding method which relies on the innate root suckering ability of a few species including dandelion, sweet potato, woody medicinals, and some legumes (Cao et al. 2023). Unfortunately, to date, neither of these methods has been adapted to achieve gene editing that is both tissue-culture and also transgene-free.

Gene editing without transgenes and **without tissue culture** is highly desirable. A few reports have achieved both, while others have reported transgenic tissue-culture free or transgene-free with tissue culture. As described in the viral delivery section, tissue-culture free gene editing can be achieved if the reagents are delivered to meristems or if viral reagents spread throughout the entire plant (T. Li et al. 2021) and (Maher et al. 2020). Alternately, transgenes or gene editing reagents can be delivered to embryonic meristems (Hamada et al. 2017; Imai et al. 2020) or axillary meristems (Manickavasagam et al. 2004; Mayavan et al. 2015). In *Arabidopsis* floral dip *Agro*-transformation, tissue-culture-free methods are routine, but limited to this and closely related species due to flower anatomy and development (Bent 2023). Two additional tissue-culture free delivery methods rely on plant cells: pollen delivery in the form of HI-EDIT (Kelliher et al. 2019)and mobile RNA delivery though grafting (Yang et al. 2023).

1.4 Supplementary Note 4: Protoplasts and Microspores

Plant protoplasts, cells with the cell wall removed, resemble animal cells and are thus amenable to many of the same transformation (aka transfection) methods, such as lipofection (Liu et al. 2020; Zhang, Iaffaldano, and Qi 2021), electroporation, and PEG/Ca^{2+}-mediated transfection, which need to bypass only the cell membrane. These transformation methods can deliver gene editing reagents to a large proportion of the protoplast cells in a tube – often over 50%, and in some optimized systems, very close to 100% (Lin et al. 2018; Woo et al. 2015; J.-F. Li et al. 2013; Yoo, Cho, and Sheen 2007). In addition, protoplasts are amenable to transfection with plasmid DNA, linear DNA, single-stranded DNA, RNA, protein, RNPs, and any combination of the above. A second advantage of protoplasts is that they are separate single cells, which can, in some

species/varieties, be regenerated into entire plants. From this perspective, highly-efficient DNA-free genome editing of protoplasts is extremely attractive for producing transgene-free edited plants (Kim et al. 2017).

However, regeneration of plants from protoplasts is not widely adopted in the industry setting. Protoplast isolation (cell wall removal) and transfection are very stressful to delicate plant cells. These stresses significantly hinder the ability to regenerate a cell wall, trigger cell division, and eventually to regenerate a healthy plant. Protocols have been developed for some varieties of some species, but are not available for the majority of species. Even in crops where these protocols have been developed, the resulting plants are often mal-formed with epigenetic defects and are typically bred to remove these defects.

Even though protoplast regeneration is uncommon, the ease of reagent delivery makes protoplasts a very useful system for rapid screening of gRNAs, nucleases, vectors, etc. After delivering reagents and allowing editing to occur, DNA can be extracted for edit detection. Alternately, researchers have also used repair of “broken” GFP or conversion of GFP and BFP to rapidly quantify gene editing in cells without needing to rely on molecular methods (Glaser, McColl, and Vadolas 2016; Zong et al. 2017; Jiang et al. 2020). However, it must be noted that non-regenerating protoplasts are non-dividing and may not be the best model for DNA repair, which varies throughout the cell cycle (non-homologous end joining more in G1, homology-directed repair more in S and G2) (Huang and Puchta 2019).

In some crops, microspores can be isolated, edited, and regenerated using methods somewhat resembling the protoplast techniques described above (Bhowmik et al. 2018). Microspore gene editing offers the advantages of single cells with high transfection efficiencies amenable to transgene-free methods. Microspores are also haploid, which can be advantageous to creating homogeneous edits, but a disadvantage if loss of allele combinations in hybrids is undesirable. To date, microspore regeneration methods are limited to a few species, but improvements continue to be made (Berenguer et al. 2021).

2 References for supplementary notes

Bent, Andrew. 2023. “Floral Dip Transformation of Arabidopsis: How, and Why Not Most Other Species?” Presented at the PlantGENE Workshop: Working towards tissue culture-free plant transformation and genome editing, Virtual. https://plantgene.atlassian.net/wiki/spaces/PH/pages/35553281/Workshops.

Berenguer, Eduardo, Elena Carneros, Yolanda Pérez-Pérez, Carmen Gil, Ana Martínez, and Pilar S Testillano. 2021. “Small Molecule Inhibitors of Mammalian GSK-3β Promote in Vitro Plant Cell Reprogramming and Somatic Embryogenesis in Crop and Forest Species.” *Journal of Experimental Botany* 72 (22): 7808–25. https://doi.org/10.1093/jxb/erab365.

Bhowmik, Pankaj, Evan Ellison, Brittany Polley, Venkatesh Bollina, Manoj Kulkarni, Kaveh Ghanbarnia, Halim Song, Caixia Gao, Daniel F. Voytas, and Sateesh Kagale. 2018. “Targeted Mutagenesis in Wheat Microspores Using CRISPR/Cas9.” *Scientific Reports* 8 (1): 6502. https://doi.org/10.1038/s41598-018-24690-8.

Cao, Xuesong, Hongtao Xie, Minglei Song, Jinghua Lu, Ping Ma, Boyu Huang, Mugui Wang, et al. 2023. “Cut–Dip–Budding Delivery System Enables Genetic Modifications in Plants without Tissue Culture.” *The Innovation* 4 (1): 100345. https://doi.org/10.1016/j.xinn.2022.100345.

Clough, S. J., and A. F. Bent. 1998. “Floral Dip: A Simplified Method for Agrobacterium-Mediated Transformation of Arabidopsis Thaliana.” *The Plant Journal: For Cell and Molecular Biology* 16 (6): 735–43. https://doi.org/10.1046/j.1365-313x.1998.00343.x.

Glaser, Astrid, Bradley McColl, and Jim Vadolas. 2016. “GFP to BFP Conversion: A Versatile Assay for the Quantification of CRISPR/Cas9-Mediated Genome Editing.” *Molecular Therapy - Nucleic Acids* 5: e334. https://doi.org/10.1038/mtna.2016.48.

Hamada, Haruyasu, Qianyan Linghu, Yozo Nagira, Ryuji Miki, Naoaki Taoka, and Ryozo Imai. 2017. “An in Planta Biolistic Method for Stable Wheat Transformation.” *Scientific Reports* 7 (1): 11443. https://doi.org/10.1038/s41598-017-11936-0.

Huang, Teng-Kuei, and Holger Puchta. 2019. “CRISPR/Cas-Mediated Gene Targeting in Plants: Finally a Turn for the Better for Homologous Recombination.” *Plant Cell Reports* 38 (4): 443–53. https://doi.org/10.1007/s00299-019-02379-0.

Imai, Ryozo, Haruyasu Hamada, Yuelin Liu, Qianyan Linghu, Yuya Kumagai, Yozo Nagira, Ryuji Miki, and Naoaki Taoka. 2020. “In Planta Particle Bombardment (IPB): A New Method for Plant Transformation and Genome Editing.” *Plant Biotechnology* 37 (2): 171–76. https://doi.org/10.5511/plantbiotechnology.20.0206a.

Jiang, Yuan-Yuan, Yi-Ping Chai, Min-Hui Lu, Xiu-Li Han, Qiupeng Lin, Yu Zhang, Qiang Zhang, et al. 2020. “Prime Editing Efficiently Generates W542L and S621I Double Mutations in Two ALS Genes in Maize.” *Genome Biology* 21 (1): 257. https://doi.org/10.1186/s13059-020-02170-5.

Kelliher, Timothy, Dakota Starr, Xiujuan Su, Guozhu Tang, Zhongying Chen, Jared Carter, Peter E. Wittich, et al. 2019. “One-Step Genome Editing of Elite Crop Germplasm during Haploid Induction.” *Nature Biotechnology* 37 (3): 287–92. https://doi.org/10.1038/s41587-019-0038-x.

Kim, Hyeran, Sang-Tae Kim, Jahee Ryu, Beum-Chang Kang, Jin-Soo Kim, and Sang-Gyu Kim. 2017. “CRISPR/Cpf1-Mediated DNA-Free Plant Genome Editing.” *Nature Communications* 8 (1): 14406. https://doi.org/10.1038/ncomms14406.

Li, Jian-Feng, Julie E. Norville, John Aach, Matthew McCormack, Dandan Zhang, Jenifer Bush, George M. Church, and Jen Sheen. 2013. “Multiplex and Homologous Recombination–Mediated Genome Editing in Arabidopsis and Nicotiana Benthamiana Using Guide RNA and Cas9.” *Nature Biotechnology* 31 (8): 688–91. https://doi.org/10.1038/nbt.2654.

Li, Tingdong, Jiacheng Hu, Yu Sun, Boshu Li, Dingliang Zhang, Wenli Li, Jinxing Liu, et al. 2021. “Highly Efficient Heritable Genome Editing in Wheat Using an RNA Virus and Bypassing Tissue Culture.” *Molecular Plant* 14 (11): 1787–98. https://doi.org/10.1016/j.molp.2021.07.010.

Lin, Choun-Sea, Chen-Tran Hsu, Ling-Hung Yang, Lan-Ying Lee, Jin-Yuan Fu, Qiao-Wei Cheng, Fu-Hui Wu, et al. 2018. “Application of Protoplast Technology to CRISPR/Cas9 Mutagenesis: From Single-Cell Mutation Detection to Mutant Plant Regeneration.” *Plant Biotechnology Journal* 16 (7): 1295–1310. https://doi.org/10.1111/pbi.12870.

Liu, Wusheng, Mary R. Rudis, Matthew H. Cheplick, Reginald J. Millwood, Jian-Ping Yang, Christine A. Ondzighi-Assoume, Garrett A. Montgomery, et al. 2020. “Lipofection-Mediated Genome Editing Using DNA-Free Delivery of the Cas9/GRNA Ribonucleoprotein into Plant Cells.” *Plant Cell Reports* 39 (2): 245–57. https://doi.org/10.1007/s00299-019-02488-w.

Maher, Michael F., Ryan A. Nasti, Macy Vollbrecht, Colby G. Starker, Matthew D. Clark, and Daniel F. Voytas. 2020. “Plant Gene Editing through de Novo Induction of Meristems.” *Nature Biotechnology* 38 (1): 84–89. https://doi.org/10.1038/s41587-019-0337-2.

Manickavasagam, M., A. Ganapathi, V.R. Anbazhagan, B. Sudhakar, N. Selvaraj, A. Vasudevan, and S. Kasthurirengan. 2004. “Agrobacterium-Mediated Genetic Transformation and Development of Herbicide-Resistant Sugarcane (Saccharum Species Hybrids) Using Axillary Buds.” *Plant Cell Reports* 23 (3). https://doi.org/10.1007/s00299-004-0794-y.

Mayavan, Subramanian, Kondeti Subramanyam, Balusamy Jaganath, Dorairaj Sathish, Markandan Manickavasagam, and Andy Ganapathi. 2015. “Agrobacterium-Mediated in Planta Genetic Transformation of Sugarcane Setts.” *Plant Cell Reports* 34 (10): 1835–48. https://doi.org/10.1007/s00299-015-1831-8.

Poddar, Snigdha, Jaclyn Tanaka, Katherine L. D. Running, Gayan K. Kariyawasam, Justin D. Faris, Timothy L. Friesen, Myeong-Je Cho, Jamie H. D. Cate, and Brian Staskawicz. 2023. “Optimization of Highly Efficient Exogenous-DNA-Free Cas9-Ribonucleoprotein Mediated Gene Editing in Disease Susceptibility Loci in Wheat (Triticum Aestivum L.).” *Frontiers in Plant Science* 13. https://www.frontiersin.org/articles/10.3389/fpls.2022.1084700.

Woo, Je Wook, Jungeun Kim, Soon Il Kwon, Claudia Corvalán, Seung Woo Cho, Hyeran Kim, Sang-Gyu Kim, Sang-Tae Kim, Sunghwa Choe, and Jin-Soo Kim. 2015. “DNA-Free Genome Editing in Plants with Preassembled CRISPR-Cas9 Ribonucleoproteins.” *Nature Biotechnology* 33 (11): 1162–64. https://doi.org/10.1038/nbt.3389.

Yang, Lei, Frank Machin, Shuangfeng Wang, Eleftheria Saplaoura, and Friedrich Kragler. 2023. “Heritable Transgene-Free Genome Editing in Plants by Grafting of Wild-Type Shoots to Transgenic Donor Rootstocks.” *Nature Biotechnology*, January, 1–10. https://doi.org/10.1038/s41587-022-01585-8.

Yoo, Sang-Dong, Young-Hee Cho, and Jen Sheen. 2007. “Arabidopsis Mesophyll Protoplasts: A Versatile Cell System for Transient Gene Expression Analysis.” *Nature Protocols* 2 (7): 1565–72. https://doi.org/10.1038/nprot.2007.199.

Zhang, Yingxiao, Brian Iaffaldano, and Yiping Qi. 2021. “CRISPR Ribonucleoprotein-Mediated Genetic Engineering in Plants.” *Plant Communications*, Technology and Applications in Plants, 2 (2): 100168. https://doi.org/10.1016/j.xplc.2021.100168.

Zong, Yuan, Yanpeng Wang, Chao Li, Rui Zhang, Kunling Chen, Yidong Ran, Jin-Long Qiu, Daowen Wang, and Caixia Gao. 2017. “Precise Base Editing in Rice, Wheat and Maize with a Cas9-Cytidine Deaminase Fusion.” *Nature Biotechnology* 35 (5): 438–40. https://doi.org/10.1038/nbt.3811.
